# Neurocognitive outcome of school-aged children with congenital heart disease who underwent cardiopulmonary bypass surgery: a systematic review protocol

**DOI:** 10.1186/s13643-019-1153-y

**Published:** 2019-10-10

**Authors:** Maria Feldmann, Cinzia Ullrich, Célina Bataillard, Walter Knirsch, Martina A. Gosteli-Peter, Beatrice Latal, Ulrike Held

**Affiliations:** 1 Child Development Centre, University Children’s Hospital Zurich, Zurich, Switzerland; 2 Children’s Research Centre, University Children’s Hospital Zurich, Zurich, Switzerland; 3 Paediatric Cardiology, Paediatric Heart Centre, University Children’s Hospital Zurich, Zurich, Switzerland; 4 Main Library, University of Zurich, Zurich, Switzerland; 5 Epidemiology, Biostatistics and Prevention Institute, Department of Biostatistics, University of Zurich, Zurich, Switzerland

**Keywords:** Congenital heart disease, Neurocognitive outcome, Intellectual outcome, IQ, Executive function, School-age

## Abstract

**Background:**

Over the past decades, survival rates of children born with congenital heart disease (CHD) have increased dramatically. Progress in prenatal diagnosis, less-invasive catheter techniques and perioperative intensive care as well as surgical techniques have led to an increased focus on extracardiac comorbidities, including potential neurodevelopmental sequelae associated with CHD. A growing body of literature reports impairments in early and school-age developmental outcome; however, there is a substantial variability in the spectrum of examined CHD types, assessment ages and applied test batteries. Furthermore, little information is available on executive function impairments in this population. Therefore, the aim of this systematic review is to determine the impact of CHD on intellectual outcome and executive functioning at school age and to determine risk factors for impaired outcomes by means of a systematic search.

**Methods:**

A systematic review of literature that reports neurodevelopmental outcome in children with CHD undergoing cardiopulmonary bypass surgery. Intelligence quotient or executive function scores will be considered primary outcomes. Databases such as Cochrane, EMBASE, MEDLINE and PsycINFO will be searched.

**Discussion:**

The results of this systematic review will summarize the current evidence on intellectual and executive function outcome after cardiopulmonary bypass surgery in school-age children with CHD. This review will thus be the basis for better patient and parental counselling and the establishment of tailored follow-up programmes and interventional trials.

**Systematic review registration:**

In accordance with the guidelines, our systematic review protocol was registered with the International Prospective Register of Systematic Reviews (PROSPERO) on January 9, 2019 (CRD42018086568). PROSPERO CRD42019118736.

## Background

Congenital heart disease (CHD) summarizes a broad array of mild to severe structural and functional congenital anomalies of the heart and great arteries. CHD occurs in about 6 infants per 1000 live births, with 1 in 1000 having a cyanotic CHD, and 2 to 3 in 1000 requiring open-heart surgery [1]. The surgical approaches range from single-stage repairs of non-cyanotic lesions (e.g. ventricular septal defect) to multiple stage palliative surgical interventions in the most complex defects of single ventricle heart diseases [1, 2].

Over the past decades, advances in cardiopulmonary bypass surgery and perioperative intensive care have lowered the mortality rates of children with CHD noticeably [2]. This led to an increasing number of patients with CHD reaching adolescence and adulthood and thus a stronger recognition of the high risk for neurodevelopmental impairment [3].

Neurodevelopmental impairment in infancy and early childhood is often mild and characterized by delayed acquisition of motor milestones and mild cognitive impairment [3] but persists into school-age and adolescence with multiple domains being simultaneously affected [13, 14]. Importantly, neurodevelopmental impairment can also be found in children with acyanotic CHD who undergo cardiopulmonary bypass surgery [14], but the risk for more severe sequelae is more pronounced with increasing complexity of the CHD [6].

Whereas the neurobehavioral profile of children with CHD in early infancy has been well characterized, studies reporting neurocognitive outcomes of children and adolescents with CHD remain scarce and comprise reports of predominantly small cohorts with heterogeneous CHD study populations. However, these studies show that during school age, impairments become more apparent with the increasing cognitive demands [6, 15].

School age is also a time when executive functions are key to social development and academic achievement [5]. Executive functions such as self-regulation and the ability to plan, solve problems and define aims are key to an independent life and represent a healthy development. This set of higher-order cognitive functions is an area of particular neurocognitive vulnerability in CHD adolescents [6]. Executive function deficits could contribute to the high prevalence of difficulties in school, which are mirrored by the high percentage of children with CHD in need for specialized educational services [16]. Behavioural difficulties such as attention deficits, hyperactivity and problems with inattention can furthermore contribute to the neurobehavioral difficulties of CHD children at school-age [17].

A number of demographic, foetal and perinatal as well as perioperative clinical risk factors for impaired neurodevelopment have been identified. Innate patient factors such as gender, birth weight, type of CHD, maternal education and genetic/extracardiac anomalies have been identified to be associated with neurodevelopmental and executive function outcome [4–7]. Furthermore, operative characteristics, such as longer cardiopulmonary bypass time and length of cardiac arrest time during the first surgery, have been identified as risk factors for poor neuropsychological outcome [4, 8]. Perioperative clinical and neurological complications, such as the need for extracorporeal membrane oxygenation, or ventricular assist devices, postoperative seizures and prolonged intensive care unit stay further contribute to the increased risk for neurodevelopmental sequelae in the CHD population [7, 8]. Thus, the underlying mechanisms leading to the neurodevelopmental sequelae are multifactorial but to date not yet fully understood [9–12].

Further research is warranted to better understand the impact of complex CHD on cognitive outcome and executive functioning at school age and the role of modifiable risk factors and will aid the clinicians to counsel and support patients and families accordingly. A systematic review of the current literature in this field will summarize the existing evidence for impaired cognitive and executive functioning, will address potential risk factors and by that provide the basis to develop future research questions.

### Objectives

We aim to systematically review the literature for long-term intellectual and executive function outcomes of school-aged children with CHD, who underwent cardiopulmonary bypass surgery in comparison to control subjects or normative reference data. We aim to further examine risk factors for poor outcome, in particular modifiable risk factors, such as operative and perioperative variables. The results of this review will be the basis for better patient and parental counselling and the establishment of tailored follow-up programmes and interventional trials.

## Methods/design

The protocol for this systematic review was developed in accordance with the Preferred Reporting Items of Systematic Reviews and Meta-Analysis for Protocols 2015 (PRISMA-P) [18] (for PRISMA-P checklist, see Additional file 1). The systematic review protocol has been registered in the International Prospective Register of Systematic Reviews (PROSPERO) on January 9, 2019 (registration number CRD42018086568).

### Eligibility criteria

#### Study design

Any original, peer-reviewed research studies including population-based register studies, retrospective and prospective cohorts and cross-sectional studies will be included. We have no restrictions to study design; however, sample size of reported results must at least be ten subjects, as the recalculation of statistical parameters is often not feasible in case series of small numbers. Abstract only publications will be excluded, but where these are identified and inclusion criteria are met, the authors will be contacted for further information on the full paper. Reviews, editorials and position papers or commentaries will be excluded, but the reference list will be screened for relevant studies.

#### Year of publication

No restriction to publication date will be applied.

#### Language

Studies in foreign languages will be translated with the help of acquainted native speakers who have a medical background, or authors will be contacted for data extraction/translation. If contacted corresponding authors do not respond within 1 month, studies will be excluded.

#### Participants

Studies reporting intellectual and/or executive function outcome of school-aged children (5–17 years) who underwent surgical repair or palliation for CHD during infancy or childhood with cardiopulmonary bypass are considered for inclusion. Studies reporting exclusively outcome of CHD children with neurologic comorbidities (e.g. due to chromosomal abnormalities or known genetic syndromal conditions) or after heart transplantation will be excluded.

Studies in which both genetic syndromal and non-syndromal patient data are reported will be included and findings of the two groups extracted and analysed separately.

#### Types of interventions, exposures and risk factors

Sociodemographic factors affecting academic performance and neurodevelopmental outcome will be considered. These include parental educational level and profession, income and environmental factors. Special educational support therapies such as early intervention as well as classroom support will be considered as exposure or confounder as appropriate. Outcomes of interventional trials will be included and extracted by intervention type. Results of the intervention group might be collated afterwards or interventional group might be accounted for in the statistical analysis.

The type of cardiac defect will be considered an exposure. Other confounders including maternal factors, neonatal factors (e.g. preterm birth, preoperative acidosis (low pH value in umbilical venous blood) or hypoxia (cyanosis)), perioperative characteristics and postoperative complications as well as pregnancy characteristics will be treated as confounders.

### Outcomes

#### Primary outcomes

Cognitive outcome in children with congenital heart disease assessed by standardized age-appropriate neuropsychological assessments and reported as intelligence quotient will be considered as primary outcome. Executive function obtained through direct assessment or questionnaire will be additionally considered as primary outcome. Studies which do not report either one of our primary outcomes will be excluded.

#### Secondary outcomes

Measures of academic achievement will be considered as secondary outcome.

### Information sources

#### Electronic database search

The following electronic databases will be searched:

- Cochrane
- Embase
- MEDLINE
- PsycInfo

The search for relevant publications will be done using subject headings (MeSH, EMTREE and PsycINDEX thesaurus) and free text words related to congenital heart disease and intellectual as well as executive function outcome in children and adolescents. The search strategy will be adapted for each database. The search will not be restricted to study design, date of publication or language.

If the full text or abstract of a reference cannot be found, authors of eligible studies will be contacted for full text or exclusion might be based on available data. If no response is received within 1 month, the study will be excluded. Reference lists of reviews, editorials and commentaries will be screened for relevant publications as stated under the section “Study design”. Journals in which five or more papers were published will be screened for additional references.

#### Search strategy

The search strategy was developed in collaboration with a health information specialist experienced in literature search for systematic reviews and the authors. Key papers were used to derive and validate the search strategy. The database search will be carried out by the information specialist. The MEDLINE search protocol is included as Additional file 2 as an example.

#### Grey literature

As we do not include grey literature, we will address reporting bias by assessing funnel plot asymmetry.

#### Selection of studies and data management

References will be stored and managed in Endnote X8 (Clarivate Analytics, Philadelphia, US). Duplicate publications will be automatically removed using Endnote X8. Remaining duplicates will be manually removed during the screening process, when identified. Management of citations throughout the fulltext screening and selection process will be supported by the web-based systematic review management programme on www.covidence.org. Two independent reviewers (CU, CB) will screen titles and abstracts of a random subsample of 200 eligible studies yielded by our search strategy. Agreement on inclusion for full-text screening of > 80 % between the two independent reviewers will be considered as good, and the remaining number of references will be divided among the reviewing authors for further screening. Full texts of potentially relevant citations will be retrieved and examined for final inclusion. Again if > 80 % agreement within a subset of the eligible studies is achieved, the remainder will not be screened in duplicate. Rationale for in- or exclusion of studies will be documented, and discussion with a third author (BL) will be carried out to find consensus. The authors will not be blinded to journal titles, authors or author affiliations during the study selection process.

We aim to identify multiple reports of the same study by juxtaposing authors and comparing the reported study populations in terms of number, year and place of recruitment as well as outcomes. Data of multiple reports of the same study will be handled as stated in the section “Data extraction”.

### Data extraction

Information will be extracted from the included studies using a digital data extraction form. The form will include demographic and clinical information on study subjects, details on applied assessment tools and reported outcome results. A draft of the data extraction form will be predefined and piloted and adapted after extracting data from the first ten included studies. For further detail, see the draft of the data extraction sheet (Additional file 3). Data extraction will be carried out by two independent authors for individual subsets of the studies or in duplicate (discrepancies solved by third party) depending on the amount of included studies and the corresponding workload. The final approach will be reported accordingly in the systematic review report. In the absence of complete outcome reports or crucial information on the study population, corresponding authors will be contacted to obtain missing information. In case of multiple reports of the same study, reporting the same follow-up time point and outcome, only the most extensive report in terms of sample size will be considered for data extraction. Conversely, two separate data extraction forms will be completed if the follow-up time points or reported outcomes differ. Information from these data extraction forms might be collated afterwards or multiple reporting will be addressed through statistical analysis. Corresponding authors might be contacted to clarify questions on overlapping reports of the same study.

### Dealing with missing data

We predefine a minimum set of information that must be extractable from the publication: age of subjects at assessment, type of CHD, cardiopulmonary bypass performed and one of the two primary outcomes reported. If the minimum dataset is not provided, corresponding authors will be contacted to retrieve missing data in order to appropriately describe the study results. If information on missing data cannot be obtained or no response of the corresponding authors is received within 1 month, the available data in each study will be used for meta-analysis and multiple imputation will be used to account for missing values if appropriate.

### Assessment of methodological quality and risk of bias of included studies

The quality of the included studies will be evaluated regarding their risk of different biases (e.g. selection bias, attrition bias, detection bias) by means of one of the SIGN checklists appropriate for study design (https://www.sign.ac.uk/checklists-and-notes.html). The overall methodological quality of the studies will be ranked as “high quality”, “acceptable” and “low quality” according to the criteria of the SIGN checklist. If a final rating is not possible due to missing information, corresponding authors will be contacted for clarification or quality will otherwise be rated as “unclear”. We do not plan to weigh study results based on the quality assessment, or to exclude studies due to low methodological quality, but will report frequencies of quality ratings and take the overall quality into consideration when discussing the results of the systematic review and meta-analysis. Risk of bias assessment will be carried out by two independent raters (MF, and additional author to be determined), and discrepancies will be solved by consulting a third party (UH).

### Assessment of heterogeneity

Heterogeneity between studies will be measured with Higgin’s *I*^2^ statistic. Higgins’s *I*^2^ statistic measures the percentage of variation between the sample estimates that is due to heterogeneity rather than to sampling error, and it can vary between 0 and 100 %. If the *I*^2^-statistic equals 0%, statistical homogeneity exists. If *I*^2^ equals 50% or more, significant heterogeneity is typically considered to be present. In this case, a random effects model will be used for meta-analysis.

### Data synthesis and evaluation of risk factors

If three or more studies report the same outcome measures, or can be grouped according to the intervention used, the results of the studies will be meta-analysed, resulting in a summary estimate. Depending on the *I*^2^-statistic, a fixed or a random effects model will be used for meta-analysis. If less than three studies are found, the results will be described and may be displayed as forest plots without summary estimate. The continuous outcome measures may be reported differently across studies, e.g. as mean and standard deviation or standard error or as median and interquartile range or range. Between-group differences may be reported as difference with confidence intervals or *t* and *p* values. If outcomes need to be recalculated from medians and interquartile ranges or ranges, the proposed formulae by Wan et al. and by Luo et al. will be used [19, 20] as recommended by the Cochrane Collaboration [21]. We will assess the moderating effect of risk factors on outcome, if risk factors were reported in sufficient detail across studies. A meta-regression approach or subgroup analysis will be used as appropriate.

### Sensitivity analyses

The reporting of the studies may be heterogeneous with respect to scales and questionnaires, statistics and time points. Homogenization may be required in order to meta-analyse the data, but it typically requires assumptions about distributions of variables, or alike. In case such assumptions are necessary before meta-analysis, a sensitivity analysis will be used to assess the effect of the assumptions on the results.

### Subgroup analyses

If possible, we will carry out subgroup analyses for the following groups:

- Type of CHD (uni- versus biventricular, cyanotic versus acyanotic, if possible further subtypes including rare types of CHD will be addressed)
- Age at surgery (< 7 days, < 1 month and other)
- Age at assessment categories (e.g. primary school age 5 to 11 years, secondary school age 12 to 17 years)
- Children receiving educational support versus no support
- Stratification by socioeconomic status

### Narrative synthesis

A narrative synthesis of the results of the systematic review will be provided, if statistical synthesis of the quantitative data is not appropriate because of a low number of studies or heterogeneous outcomes that cannot be pooled. In this case, a predeveloped narrative synthesis method (such as Popay et al. [22]) will be used. Results will be presented in descriptive paragraphs and might be supplemented by visual displays and tables as suitable. The most appropriate methods will be determined during the review process and outlined in the final manuscript.

### Assessment of meta-bias

Publication bias will be assessed by the graphical method of funnel plot, and the presence of bias will be visually inspected and statistically tested using the Egger test [23].

### Confidence in cumulative evidence

To judge the confidence in the resulting body of evidence, the strength of the evidence will be assessed using the Grading of Recommendations Assessment (GRADE) [24].

### Amendments

In the event of protocol amendments, the date of each amendment will be provided with a description of the change and the rationale. This information will be added in tabular form to the final report and manuscript of the systematic review.

## Discussion

The results of this systematic review will contribute to a better understanding of the impact of CHD on cognitive and executive function outcomes at school and adolescent age. In particular, modifiable surgical and perioperative risk factors may be identified through this systematic review and may help to improve clinical management. The summary of the current literature will moreover aid clinicians to better understand the impact of different factors on outcome and thus help to counsel patients and their families. This information will be the basis for tailored follow-up programmes. Moreover, it will help to stratify risk groups to develop and provide early pharmacological and non-pharmacological intervention therapies. Finally, it will aid researchers to detect knowledge gaps and will help to pose future research questions.

With the here outlined methodological approach to our systematic review, which is in compliance with the PRISMA-P reporting guidelines, we aim to provide methodological transparency and clarity that is crucial for future reproducibility and enhance the value and strength of the evidence we will obtain from the results of our systematic review and meta-analysis.

## Supplementary information

**Additional File 1:** PRISMA-P + checklist_10012018.docx. Prisma-P checklist (DOCX 32 kb)

**Additional File 2:** _20190819_revised.docx. Medline search strategy (DOCX 21 kb)

**Additional File 3:** _20190819_revised.docx. Draft of data extraction sheet (DOCX 15 kb)

## Data Availability

Not applicable.

## Abbreviations

CHD: Congenital heart disease
PRISMA-P: Preferred Reporting Items of Systematic Reviews and Meta-Analysis for Protocols
