# Supplementary material for: Neurocognitive outcome of school-aged children with congenital heart disease who underwent cardiopulmonary bypass surgery: a systematic review protocol
Source: Syst Rev. 2019 Oct 10;8:236. doi: 10.1186/s13643-019-1153-y (PMC6787965; doi:10.1186/s13643-019-1153-y)
Supplement: Supplementary file 2 — Additional File 2: _20190819_revised.docx. Medline search strategy (DOCX 21 kb) [file 13643_2019_1153_MOESM2_ESM.docx]

# Additional File 2

## **Ovid MEDLINE(R) and Epub Ahead of Print, In-Process & Other Non-Indexed Citations and Daily** 1946 to July 09, 2019

## Search Strategy:

| **#** | **Searches** |
| --- | --- |
| 1 | exp Heart Defects, Congenital/ or exp Heart Diseases/cn or ((congenital* or hereditary or inborn) and ((heart* or cardiac* or coronary or septal* or aortopulmonary or aorticopulmonary or atrial or ventricular or intraventricular) adj3 (defect* or disease* or malformation* or abnormal* or anomal*))).ti,ab. or (digeorge adj1 (syndrome* or anomal* or sequenc*)).ti,ab. or (transpos* adj3 (arteries or artery or vessel*)).ti,ab. or (alagille adj2 syndrome).ti,ab. or ("arteriohepatic dysplasia*" or "gonadal dysgenesis" or "subdivided left atrium*").ti,ab. or ((cardiovertebral or "pharyngeal pouch" or "thymic aplasia" or "conotruncal anomaly face" or turner* or noonan or barth or velo* or kartagener* or siewert* or scimitar or lutembacher* or leopard or "multiple lentigines" or marfan*) adj3 syndrome*).ti,ab. or ("hepatic hypoplasia" or "arteriohepatic dysplasia*" or "bicuspid aortic valve").ti,ab. or (taussig* adj2 anomal*).ti,ab. or ((pulmon* or aortic or subaortic or valve or mitral) adj1 stenosis).ti,ab. or ((aortic or aorta*) adj3 coarctation*).ti,ab. or (ventricular adj2 dysplasia*).ti,ab. or ("cor triatriatum" or cortriatriatum or "triatrial heart*").ti,ab. or ("myocardial bridging*" or "crisscross heart*" or "criss‐cross heart*").ti,ab. or (dextrocardia* or "kartagener* triad" or "primary ciliary dyskinesia").ti,ab. or ("patent ductus arteriosus" or "anomalous pulmonary venous connection" or "double inlet left ventricle" or "double outlet right ventricle" or "interrupted aortic arch").ti,ab. or ("ebstein* anomaly" or "ebstein* malformation*" or "ectopia cordis").ti,ab. or (eisenmenger* adj1 (complex or syndrome)).ti,ab. or ("persistent truncus arteriosus" or "persistent ostium primum").ti,ab. or ("endocardial cushion defect*" or "atrioventricular canal").ti,ab. or "foramen oval*".ti,ab. or (heart adj3 hypoplas*).ti,ab. or ((noncompaction or "non compaction") adj3 "ventricular myocardium").ti,ab. or levocardia.ti,ab. or (((tetralogy or trilogy or syndrome) adj2 fallot*) or cantrell* or shon?s).ti,ab. or ((tricuspid or valve or pulmonary) adj1 atresia*).ti,ab. or ("absent right atrioventricular connection" or "single ventricle physiology" or GUCH or "cavopulmonary connection").ti,ab. or ((bonnevie adj2 (syndrome* or status)) or "polynesian bronchiectas*").ti,ab. |
| 2 | cardiac surgical procedures/ or arterial switch operation/ or cardiac valve annuloplasty/ or mitral valve annuloplasty/ or heart arrest, induced/ or circulatory arrest, deep hypothermia induced/ or heart bypass, right/ or fontan procedure/ or norwood procedures/ or "cardiopulmonary bypass"/ or exp Heart Defects, Congenital/su or ((heart or cardia* or myocardia* or cardiopulmonary or corrective or reparative or repair* or bypass) adj3 (surgery or operation* or procedure* or method* or technique* or bypass)).ti,ab. or (surg* adj3 correct*).ti,ab. |
| 3 | 1 and 2 |
| 4 | (((warden or rastelli or damus or kaye or dansel or ebstein or glenn or ross or bentall or fontan or norwood or blalock or taussig or aorto-pulmonary or "arterial switch" or "atrial switch" or "double switch" or fallot or jatene or mustard) adj3 (procedure* or surgery or operation* or repair* or closure* or conduit* or connection* or anastomosis or shunt or technique*)) or ((dextrotransposition or transposition or D-transposition) adj3 (arteries or vessels))).ti,ab. |
| 5 | 3 or 4 |
| 6 | exp child/ or exp adolescent/ or (child or children or minor* or teen* or juvenile* or adolescent*).ti,ab,kf. or ((exp pediatrics/ or (pediatric* or paediatric*).ti,ab,kf.) not (exp Infant/ not child/)) |
| 7 | exp Memory Disorders/ or exp Executive Function/ or exp Memory/ or exp psychological tests/ or exp neuropsychological tests/ or exp Intelligence Tests/ or exp Intelligence/ or exp Cognition Disorders/ or exp Cognition/ or exp neurocognitive disorders/ or exp neurodevelopmental disorders/ or (cognitive or cognition or IQ or intellectual or intelligence or executive or memory or neuropsycholog* or neurodevelop* or neurocognit* or neurobehav* or neuro- psycholog* or neuro-develop* or neuro-cognit* or neuro-behav*).ti,ab. or (developmental adj3 (outcome* or evaluation* or problem* or delay*)).ti,ab. or ((motor or psychomotor or school or work or language or daily or everyday) adj3 (performance or abilities or proficiency or skills)).ti,ab. |
| 8 | 5 and 6 and 7 |
