# Supplementary material for: Neurocognitive outcome of school-aged children with congenital heart disease who underwent cardiopulmonary bypass surgery: a systematic review protocol
Source: Syst Rev. 2019 Oct 10;8:236. doi: 10.1186/s13643-019-1153-y (PMC6787965; doi:10.1186/s13643-019-1153-y)
Supplement: Supplementary file 3 — Additional File 3: _20190819_revised.docx. Draft of data extraction sheet (DOCX 15 kb) [file 13643_2019_1153_MOESM3_ESM.docx]

**Additional File 3**

## Draft of data extraction sheet: predefined items

| **Author** |
| --- |
| **Year** |
| **Journal** |
| **Title** |
| **Subgroup (new column for each reported CHD subtype and controls)** |
| **Study Design** |
| **Participants** |
| Subjects (number) |
| Age at assessment (mean/median; distribution; unit distribution; unit age) |
| Recruitment period (year-year) |
| Country of enrolment (free text) |
| Suspicion of multiple reporting of same cohort (yes/no) |
| **Perioperative characteristics** |
| Preoperative baseline characteristics |
| Birth weight (mean/median in gram) |
| Gestational age (weeks) |
| Prematurity (<37wk) (number) |
| Apgar score at 5 min (mean/median) |
| Sex (number male) |
| Race (number white) |
| Prenatal diagnosis (number yes) |
| Maternal Age (mean/median in years) |
| Socioeconomic status (mean/median value; assessment scale) |
| Cyanotic heart defect (number) |
| Without CPB (number) |
| Univentricular (number) |
| Surgical data |
| Weight at first surgery (mean/median; unit) |
| Age at first surgery (mean/median; unit) |
| Circulatory arrest time (mean/median in min) |
| Total bypass time (mean/median in min) |
| Lowest temperature during first surgery (mean/median in °C) |
| Length of ICU stay after first surgery (mean/median in d) |
| Length of hospital stay (mean/median in d) |
| Antegrade cerebral perfusion (mean/median in min) |
| Postoperative neurological outcomes |
| Postoperative seizures (number) |
| **Follow-up data** |
| Follow-up rate (%) |
| Head circumference (cm or z-score) |
| Weight (kg or z-score) |
| Height (cm or z-score) |
| Neurodevelopmental outcomes |
| Type of test (free text) |
| Total IQ (mean/median; distribution; unit distribution) |
| Verbal IQ (mean/median; distribution; unit distribution) |
| Performance IQ (mean/median; distribution; unit distribution) |
| Processing speed IQ (mean/median; distribution; unit distribution) |
| Working memory IQ (mean/median; distribution; unit distribution) |
| Neurologic examination: |
| No abnormality (number) |
| Any abnormality (number) |
| Cerebral palsy (number) |
| Academic achievement |
| Assessment of academic achievement (yes/no) |
| Type of assessment (free text) |
| Test result (mean/median) |
| Executive function assessment |
| Executive function assessed (yes/no) |
| Type of EF Test (free text) |
| EF Results (mean/median; distribution; unit distribution; unit) |
| BRIEF reported (yes/no) |
| BRIEF (parent-reported) total score (mean/median; distribution; unit distribution) |
| BRIEF (self-reported) total score (mean/median; distribution; unit distribution) |
